# Improving perceptions of healthy food affordability: results from a pilot intervention

**DOI:** 10.1186/1479-5868-11-33

**Published:** 2014-03-10

**Authors:** Lauren K Williams, Gavin Abbott, Lukar E Thornton, Anthony Worsley, Kylie Ball, David Crawford

**Affiliations:** 1 Centre for Physical Activity and Nutrition Research, Deakin University, 221 Burwood Highway, Burwood, Victoria 3125, Australia; 2 Murdoch Childrens Research Institute, Flemington Road, Parkville, Victoria 3052, Australia

**Keywords:** Diet, Fruit and vegetable consumption, Mothers, Food affordability

## Abstract

**Background:**

Despite strong empirical support for the association between perceived food affordability and dietary intake amongst families with a lower socioeconomic position (SEP), there is limited evidence of the most effective strategies for promoting more positive perceptions of healthy food affordability among this group. This paper reports findings from a pilot intervention that aimed to improve perceptions of healthy food affordability amongst mothers.

**Findings:**

Participants were 66 mothers who were the parents of children recruited from primary schools located in socioeconomically disadvantaged suburbs. Intervention group participants viewed a slideshow focussed on healthy snack food affordability that illustrated cheaper healthier alternatives to common snack foods as well as food budgeting tips and price comparison education. A mixed between-within ANCOVA was conducted to examine group differences in perceived affordability of healthy food across three time points. Results revealed no difference in perceived affordability of healthy food between the two groups at baseline whereas at post-intervention and follow-up, mothers in the intervention group perceived healthy food as more affordable than the control group.

**Conclusions:**

Focussing on education-based interventions to improve perceptions of healthy food affordability may be a promising approach that complements existing nutrition promotion strategies.

## Introduction

Amongst families of low socio-economic position (SEP), cost is cited as one of the most common barriers to both maternal and child healthy eating [1-3], and there is evidence that when funds are limited, more affordable options high in sugar and fat are often consumed, resulting in poor nutrition and increased obesity risk [4,5]. While it is noted that actual food prices impact food decision making, changing food prices can be difficult and expensive. Whilst nutrition promotion efforts should continue to focus on making healthy food more accessible and affordable, the psychological mechanisms attached to food choice and consumption, such as perceptions of healthy food affordability, are also important and should not be overlooked [6]. Intervention approaches targeting food cost, such as ‘fat tax’ or food subsidies, are often touted as solutions to epidemics of poor eating, yet may be less effective if healthy foods are perceived as high cost relative to less healthy options. Despite empirical support for the association between perceived food affordability and dietary intake amongst low SEP families [7], there is limited evidence of the most effective strategies for promoting more positive perceptions of healthy food affordability among this group. Improving perceptions of healthy food affordability may offer a feasible, population-based preventive-orientated approach to nutrition promotion. The purpose of this paper is to report findings from a pilot intervention that exposed a group of mothers to a psycho-education based slideshow depicting healthy affordable snack food, with the aim of improving their perceptions of healthy food affordability. The minimal psycho-educational approach was chosen on the basis that it is feasible, low-cost and addresses perceptions of lack of affordability of healthy food which is a commonly reported barrier to healthy eating [1,6].

## Methods

### Participants & procedure

Participants were 66 mothers who were the parents of children recruited from primary schools drawn from a random sample of 33 suburbs ranked in the lowest quintile of relative neighbourhood disadvantage in the state of Victoria. Our sample size yielded sufficient power based on a priori sample size calculations that incorporated results from previous studies, 90% power, alpha 0.05 and adjusting for a time effect. Senior staff from forty primary schools were approached in 2011, of which 13 consented researchers to advertise their study via school newsletters and study flyers. The number of mothers per school ranged from 1-13 and all mothers resided in the same suburb as the school their child attended. Consenting participants were randomised to either intervention or control group. Both groups completed two surveys and viewed a slideshow during a one-on-one session with the researcher in a private room located on school grounds. A survey, which measured sociodemographic characteristics and perceived affordability of healthy food, was completed three times in total; 1) prior to viewing the slideshow (Time 1), 2) immediately after viewing the slideshow (Time 2) and 3) four weeks after viewing the slideshow (Time 3). Completion of the survey at time one and two occurred on the same day as the intervention. The total sample of participants at time two was 57 (83.8% retention rate).

Ethics approval for this study was obtained from the Deakin University Human Ethics Advisory Group, Faculty of Health [#HEAG-H 71_2011].

### Measures

#### Intervention

Participants viewed an automated slideshow focussed on healthy snack food affordability. A slideshow format was selected to ensure delivery consistency across groups (i.e. the same slideshow can be used multiple times with 100% consistency in content), to minimise cost, and to test the utility of the slideshow format as a method to potentially maximise resources and therefore, reach for future replication. Snack foods were selected given they comprise a significant proportion of overall consumption, are provided in high quantities in Australian children’s lunchboxes and are foods associated with poor nutrition and obesity [8]. Selected snack foods for the slideshow were based on a) common snack foods consumed by Australian children [8] and b) fruit and vegetables that can be readily consumed as snacks. The slideshow content was based on resources from the Western Australia Food Cent$ program [9] and local Government food security program resources. Pilot testing of the slideshow was conducted with a convenience separate sample of mothers prior to delivery. Pilot participants provided feedback (e.g. slideshow content, timing, visual appeal etc.) on the slideshow. The slideshow included illustrated examples of cheaper healthier alternatives to common snack foods as well as food budgeting tips and price comparison education. For example, part one of the slideshow included a voice recording with illustrations of paired snack food items whereby the viewer was asked which of two items were cheaper (e.g. a snack bar or an apple), followed by the prices to reveal the healthier item was cheaper. Participants were also shown in the slideshow how to provide healthy snacks by buying in bulk (e.g. 1 kg yoghurt versus 6 × 200 ml containers) or using homemade items (e.g. popcorn). The price comparison education component of the slideshow illustrated how to compare prices p/kilogram of healthy and less healthy items. Participants were also shown how much money they could save by changing to healthy and cheaper snack food options. The slideshow lasted approximately 10 minutes. Control group participants were shown a 10 minute slideshow on bicycle safety and completed the same survey assessments as the intervention group.

#### Perceived affordability of healthy food

Perceived healthy food affordability was assessed using a scale devised for the current study using items adapted from previous studies conducted by the authors [7] and others [10-12]. The perceived healthy food affordability scale comprised the following: a) 5 items designed to assess attitudes towards the cost of healthy food (e.g. “I feel that healthy snack food options are too expensive”) and b) 5 items designed to assess purchasing behaviour relevant to the cost of healthy food (e.g. ‘Sometimes my family cannot afford to buy healthy and nutritious food’). Each item was scored on a six-point scale ranging from ‘Strongly disagree’ (1) to ‘Strongly agree’ (6). A total scale score for perceived affordability of healthy food was produced by summing the 10 items, with a maximum range of 10-60. Lower scores indicate the perception that healthy foods are more affordable. Using Cronbach’s alpha, internal reliability of the scale at each time point was 0.91, 0.93, and 0.93 for baseline, post-intervention, and follow-up respectively.

#### Sociodemographic characteristics (Covariates)

Mothers provided information regarding their age, highest level of education, weekly household income, and number of children.

### Statistical analysis

To minimize the impact of missing data at time 3, an intention to treat approach was applied and mothers who were missing data for perceived affordability of healthy food at T3 (n = 12) had missing scores replaced with their baseline score. Although 68 mothers participated at baseline, one mother missing baseline outcome data and one mother missing education data were excluded from analyses, leaving a final sample of 66 mothers.

T-tests and chi-square tests were used to assess group differences in baseline sociodemographic characteristics. To assess the effect of the intervention on participants’ perceived affordability of healthy food, a mixed between-within (time X group) ANCOVA was conducted, with perceived affordability of healthy food across the three time points as the outcome, and intervention group as a between subjects predictor. Participants’ age, highest level of education, household income, and number of children were included as covariates in the model. All analyses were conducted using IBM SPSS Statistics 20.

## Findings

**Figure 1:**
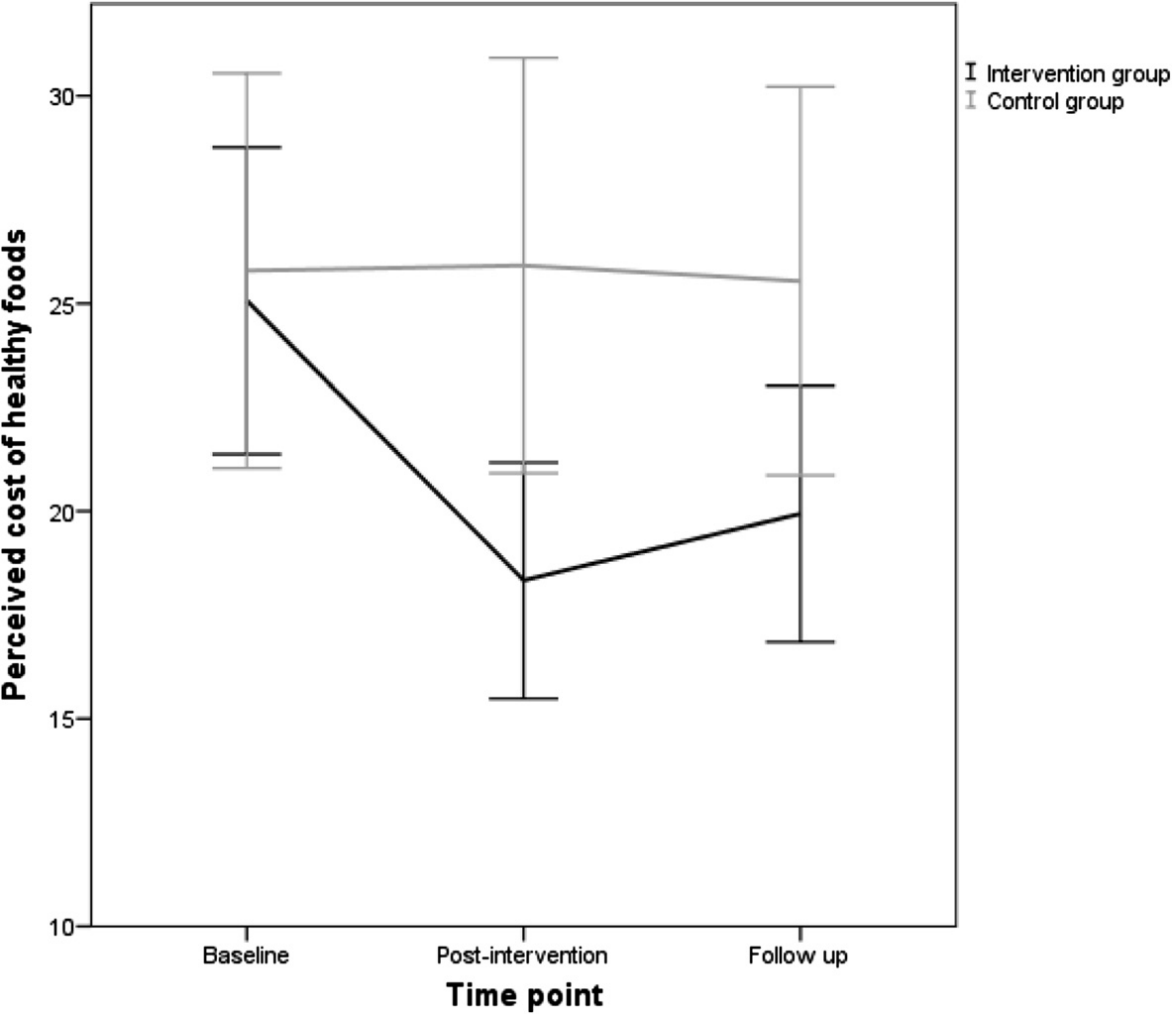
**Perceived cost of healthy foods for intervention and control groups at three time points.** Error bars represent 95% confidence intervals.

Sociodemographic characteristics of the sample are presented in Table 1. A significant main effect for group (*F*[1] = 4.960, *p* = .030, *ηρ*^2^ = .076) and time × group interaction (*F*[2] = 5.339, *p* = .006, *ηρ*^2^ = .082) was observed with a medium effect size highlighting a difference in perceived affordability of healthy food between the intervention and control group that varied by time of intervention delivery. Specifically, post-hoc tests revealed no difference in perceived affordability of healthy food between the two groups at baseline whereas at post-intervention and follow-up, mothers in the intervention group perceived healthy food as more affordable than the control group (Table 1 and Figure 1).

**Table 1:** Sociodemographic characteristics and group differences in mean (SD) of perceived affordability of healthy food at each time point

	**Whole sample (n = 66)**	**Control (n = 24)**	**Intervention (n = 42)**	**p**^**a**^
	**%**	**%**	**%**	
Age: m*ean (SD)*	40.2 (6.1)	40.1 (6.3)	40.5 (5.9)	.802
Education				.063
Not tertiary	60.6	45.8	69.0	
Tertiary	39.4	54.2	31.0	
Household income				.879
Low ($0-999/week)	24.2	20.8	26.2	
Medium ($10000-1999/week)	28.2	33.3	26.2	
High ($2000+/week)	27.3	29.2	26.2	
Undisclosed	19.7	16.7	21.4	
Number of children				.625
One	22.7	29.2	19.0	
Two	57.6	54.2	59.5	
Three or more	19.7	16.7	21.4	
	Mean (SD)	Mean (SD)	Mean (SD)	p^b^
T1 (baseline) perceived affordability of healthy food: m*ean (SD)*	25.3 (11.6)	25.8 (11.3)	25.1 (11.9)	.587
T2 perceived affordability of healthy food: m*ean (SD)*	21.1 (10.8)	25.9 (11.8)	18.3 (9.1)	.004
T3 perceived affordability of healthy food: m*ean (SD)*	22.0 (10.6)	25.5 (11.1)	19.9 (9.9)	.009

^a^Differences between control and intervention groups were assessed by t-tests for age, and chi-square tests for categorical characteristics.

^b^Differences between control and intervention groups were assessed using separate one-way ANCOVAs with perceived affordability of healthy foods at each time point (outcome) with group as the predictor, and adjusting for age, highest level of education, household income, and number of children.

## Discussion

The results from this pilot study provide preliminary support for an education-based intervention targeting perceptions of healthy food affordability. Compared to mothers in the control group, mothers in the intervention group perceived healthy food as more affordable post-intervention, a change that was sustained at follow-up.

Despite the empirical impetus on perceived healthy food affordability as a key mechanism for consumption of a healthy diet, nutrition promotion interventions targeting cost have focused primarily on ‘upstream’ fiscal food policies, namely providing financial assistance or reducing the prices of healthier foods in relation to less healthy alternatives [13]. Whilst there is evidence that price reductions can increase healthy food purchasing, these results are tempered by evidence that a) unintended compensatory purchasing of high calorie foods can result, being counter to health, and b) increased purchasing typically declines substantially over time [14]. Although cost is very important, it is only one component of successful nutrition-related behaviour change, and is a difficult and expensive component to target, particularly if the price reduction needed to increase healthy food purchasing is substantial [15]. Although it is not impossible that the negative side effects of pricing strategies may also occur when targeting perceptions of cost, given that major ongoing support and resources are required to sustain price-reduction strategies, focussing on education based interventions to improve perceptions of healthy food affordability may compliment evidence based price reduction strategies and provide a feasible long-term solution to nutrition promotion strategies amongst families of low SEP.

Limitations of the study include the small sample size, potential for socially desirable responses from the intervention group, potential effect of discrepant education levels in the intervention vs control group, and loss of participants at follow-up. Nonetheless, the findings provide tentative support that a brief education-based intervention may reduce mothers’ perceptions of the costs of healthy food. Future research efforts should focus on establishing whether the effects observed in the current study can be generalized to a larger sample and whether the materials can be successfully translated into a format easily accessed by larger numbers (e.g. DVD, advertisements etc). Further research of this nature should also include cost-effectiveness analyses to ascertain the cost-related feasibility of these formats. Further, given our study did not assess the impact of improved perceived healthy food affordability on purchasing behaviours and dietary intake, future research is needed to examine the effectiveness of the intervention on healthy food consumption amongst mothers and children residing in socioeconomically disadvantaged neighbourhoods. Nutrition promotion interventions should incorporate strategies that focus on improvements to perceptions of healthy food affordability as a preventively-orientated adjunct approach relative to more resource-intensive methods targeting food cost alone.

## Competing interests

The authors declare that there are no competing interests.

## Authors’ contribution

LW designed and coordinated the study and drafted the manuscript. GA conducted the statistical analyses. LT, TW, KB & DC contributed to the study conception and design. GA, LT, TW, KB & DC contributed to the conceptualisation and writing of the manuscript. All authors read and approved the final manuscript.

## References

[B1] Pollard J Kirk SF Cade JE Factors affecting food choice in relation to fruit and vegetable intake: a review Nutr Res Rev 2002 15 373 387 10.1079/NRR200244 19087412

[B2] Williams LK Abbott G Crawford D Ball K Associations between mothers’ perceptions of the cost of fruit and vegetables and children’s diets: will children pay the price? Eur J Clin Nutr 2012 66 276 278 10.1038/ejcn.2011.164 21952694

[B3] NiMurchu C Eyles H Dixon R Matoe L Teevale T Meagher-Lundberg P Economic incentives to promote healthier food purchases: exploring acceptability and key factors for success Health Promot Int 2012 27 331 341 10.1093/heapro/dar042 21742702

[B4] Rydén PJ Hagfors L Diet cost, diet quality and socioeconomic position: how are they related and what contributes to differences in diet costs? Public Health Nutr 2011 14 1680 1692 10.1017/S1368980010003642 21255480

[B5] Beydoun MA Powell LM Chen X Wang Y Food prices are associated with dietary quality, fast food consumption, and body mass index among U.S. children and adolescents J Nutr 2011 141 304 311 10.3945/jn.110.132613 21178080 PMC3021450

[B6] Inglis V Ball K Crawford D Socioeconomic variations in women’s diet: what is the role of perceptions of the local food environment? J Epidemiol Community Health 2008 62 191 197 10.1136/jech.2006.059253 18272732

[B7] Williams L Ball K Crawford D Why do some socioeconomically disadvantaged women eat better than others? An investigation of the personal, social and environmental correlates of fruit and vegetable consumption Appetite 2010 55 441 446 10.1016/j.appet.2010.08.004 20728488

[B8] Sanigorski A Bell AC Kremer PJ Swinburn BA Lunchbox contents of Australian school children: room for improvement Eur J Clin Nutr 2005 59 1310 1316 10.1038/sj.ejcn.1602244 16034359

[B9] Pollard C Miller M Woodman RJ Meng R Binns C Changes in knowledge, beliefs, and behaviors related o fruit and vegetable consumption among Western Australian adults from 1995 to 2004 Am J Public Health 2008 99 355 361 19059859 10.2105/AJPH.2007.131367 PMC2622794

[B10] Turrell G Kavanagh AM Socio-economic pathways to diet: modelling the association between socio-economic position and food purchasing behaviour Public Health Nutr 2006 9 375 383 10.1079/PHN2006850 16684390

[B11] Bihan H Castetbon K Mejean C Peneau S Pelabon L Jellouli F Le Clesiau H Hercberg S Sociodemographic factors and attitudes toward food affordability and health are associated with fruit and vegetable consumption in a low-income French population J Nutr 2010 140 823 830 10.3945/jn.109.118273 20181785

[B12] Dibsdall LA Lambert N Bobbin RF Frewer LJ Low-income consumers’ attitudes and behaviour towards access, availability and motivation to eat fruit and vegetables Public Health Nutr 2003 6 159 168 12675958 10.1079/PHN2002412

[B13] Ball K McNaughton SA Ni Mhurchu C Andrianopoulos N Inglis V McNeilly B Le HND Leslie D Pollard C Crawford D Supermarket healthy eating for life (SHELf): protocol of a randomised controlled trial promoting healthy food and beverage consumption though price reduction and skill-building strategies BMC Public Health 2011 11 715 10.1186/1471-2458-11-715 21936957 PMC3186753

[B14] Ni Mhurchu C Blakely T Jiang Y Eyles HC Rodger A Effects of price discounts and tailored nutrition education on supermarket purchases: a randomized controlled trial Am J Clin Nutr 2010 91 736 747 10.3945/ajcn.2009.28742 20042528

[B15] Waterlander WE de Boer MR Schuit AJ Seidell JC Steenhuis IH Price discounts significantly enhance fruit and vegetable purchases when combined with nutrition education: a randomized controlled supermarket trial Am J Clin Nutr 2013 97 886 895 10.3945/ajcn.112.041632 23446898

